# *HMGA1-pseudogene7* transgenic mice develop B cell lymphomas

**DOI:** 10.1038/s41598-020-62974-0

**Published:** 2020-04-27

**Authors:** Marco De Martino, Davide De Biase, Floriana Forzati, Sara Carmela Credendino, Giuseppe Palma, Antonio Barbieri, Claudio Arra, Orlando Paciello, Eugenio Gaudio, Maurilio Ponzoni, Gabriella De Vita, Paolo Chieffi, Francesco Bertoni, Alfredo Fusco, Francesco Esposito

**Affiliations:** 1 Istituto di Endocrinologia ed Oncologia Sperimentale - CNR c/o Dipartimento di Medicina Molecolare e Biotecnologie Mediche, Università degli Studi di Napoli “Federico II”, Naples, Italy; 2 Department of Veterinary Medicine and Animal Production, University of Naples Federico II, Naples, Italy; 3 S.S.D. Sperimentazione Animale, Istituto Nazionale Tumori, IRCCS, Fondazione Pascale, Naples, Italy; 4 Institute of Oncology Research, Faculty of Biomedical Sciences, USI, Bellinzona, Switzerland; 5 Vita-Salute San Raffaele University & Pathology Unit, IRCCS San Raffaele Scientific Institute, Milan, Italy; 6 Department of Psychology, University of Campania “L. Vanvitelli”, Caserta, Italy

**Keywords:** Cancer epigenetics, Oncogenes

## Abstract

We have recently identified and characterized two pseudogenes (*HMGA1P6* and *HMGA1P7*) of the *HMGA1* gene, which has a critical role in malignant cell transformation and cancer progression. *HMGA1P6* and *HMGAP17* act as microRNA decoy for *HMGA1* and other cancer-related genes upregulating their protein levels. We have previously shown that they are upregulated in several human carcinomas, and their expression positively correlates with a poor prognosis and an advanced cancer stage. To evaluate *in vivo* oncogenic activity of *HMGA1* pseudogenes, we have generated a *HMGA1P7* transgenic mouse line overexpressing this pseudogene. By a mean age of 12 months, about 50% of the transgenic mice developed splenomegaly and accumulation of lymphoid cells in several body compartments. For these mice FACS and immunohistochemical analyses suggested the diagnosis of B-cell lymphoma that was further supported by clonality analyses and RNA expression profile of the pathological tissues of the *HMGA1P7* transgenic tissues. Therefore, these results clearly demonstrate the oncogenic activity of *HMGA1* pseudogenes *in vivo*.

## Introduction

Many evidences indicate that long non-coding RNAs (lncRNAs) are key modulators of different biological phenomena. Given this scenario, it is predictable that deregulated expression and aberrant role of lncRNAs are involved in the development of several diseases including cancer^1^. Among lncRNAs, pseudogenes, a subgroup of genes that arises from protein-coding genes that have lost the capacity to produce proteins, have been considered for long time as non-functional genomic junk^1^. However, recent studies have unveiled important functions of pseudogenes in the regulation of the expression of the parental genes. Indeed, the majority of the identified pseudogenes has high sequence homology with their protein-coding parental counterparts, enabling them to take part in post-transcriptional control of their parental genes. The regulation of parental gene relies on several mechanisms: (i) the generation of endogenous short interfering RNAs (siRNAs)^2,3^; (ii) the engagement of regulatory proteins on the parental gene by pseudogene RNAs to control gene expression and chromatin remodelling^4,5^; (iii) the ability of the pseudogenes to compete with the parental genes for RNA-binding proteins and the translation machinery^6–8^; (iiii) the ability of pseudogenes to compete with their parental genes for a common pool of shared microRNAs (miRNAs)^9^ through the high sequence homology of the 3′ Untranslated region (UTR), thus regulating each other expression as competitive endogenous RNAs (ceRNAs)^10^.

The HMGA protein family includes the HMGA1a, HMGA1b and HMGA2 members^11^. The first two are coded for by the same gene through an alternative splicing. They have no transcriptional activity *per se*, but, modifying the chromatin architecture, they are able to positively or negatively regulate the expression of several genes, particularly those involved in cancer progression^11,12^. Consistently, these proteins are expressed at very low levels in normal adult tissues, but are abundant in almost all the human malignant neoplasms^11^, and their expression significantly correlates with the capability of cancer cells to metastatize and a patient poor prognosis^13–15^. Moreover, *in vitro* and *in vivo* models support a causal role of the HMGA proteins in cell transformation and cancer development^11,16,17^.

We have recently identified two human *HMGA1* processed pseudogenes (*HMGA1P6* and *HMGA1P7*) that are not present in mouse genome. *HMGA1P6* and *HMGA1P7* can compete with *HMGA1* for miRNA binding, leading to the upregulation of HMGA1 cellular levels, thereby enhancing the expression of cell malignant features^18–23^. The overexpression of these *HMGA1* pseudogenes (*HMGA1Ps*) also increases the levels of *HMGA2* and other cancer-related genes, such as *EZH2* and *VEGF*, by inhibiting the suppression of their synthesis^18^. Noteworthy, *HMGA1Ps* were found overexpressed in several human cancer types supporting their involvement in carcinogenesis^18,20–23^. To investigate the role of *HMGA1* pseudogenes overexpression *in vivo*, we generated transgenic mouse model overexpressing *HMGA1P7* (*HMGA1P7*-TG)^18,22–24^. Mouse Embryonic Fibroblasts (MEFs) derived from *HMGA1* pseudogene transgenic mice showed a higher growth rate and a later onset of senescence than the wild-type (WT) counterpart^18^.

Here, we report that *HMGA1* pseudogene transgenic mice develop haematological neoplasia characterized by monoclonal B-cell populations, most of them diagnosed as large B-cell lymphoma. These results validate the oncogenic role of the *HMGA1* pseudogenes^18^.

## Results

### *HMGA1P7* transgenic mice develop lymphoproliferative lesions

**Figure 1:**
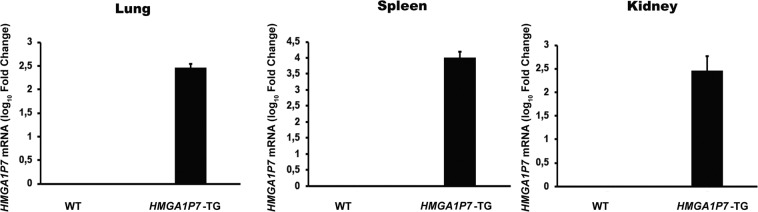
Analysis of *HMGA1P7* expression in transgenic mice qRT-PCR analysis of total RNA from lungs, spleens and kidneys of WT (n = 3) and *HMGA1P7* (n = 3) transgenic mice. The error bars represent mean ± SD.

Transgenic mice carrying the *HMGA1P7* gene were generated by the injection of the transgene into C57BL/6N derived-zygotes and, then transferred into pseudo-pregnant as previously described^18^. The expression of the *HMGA1P7* was assessed in lungs, spleens and kidneys explanted from transgenic mice (Fig. 1).

**Figure 2:**
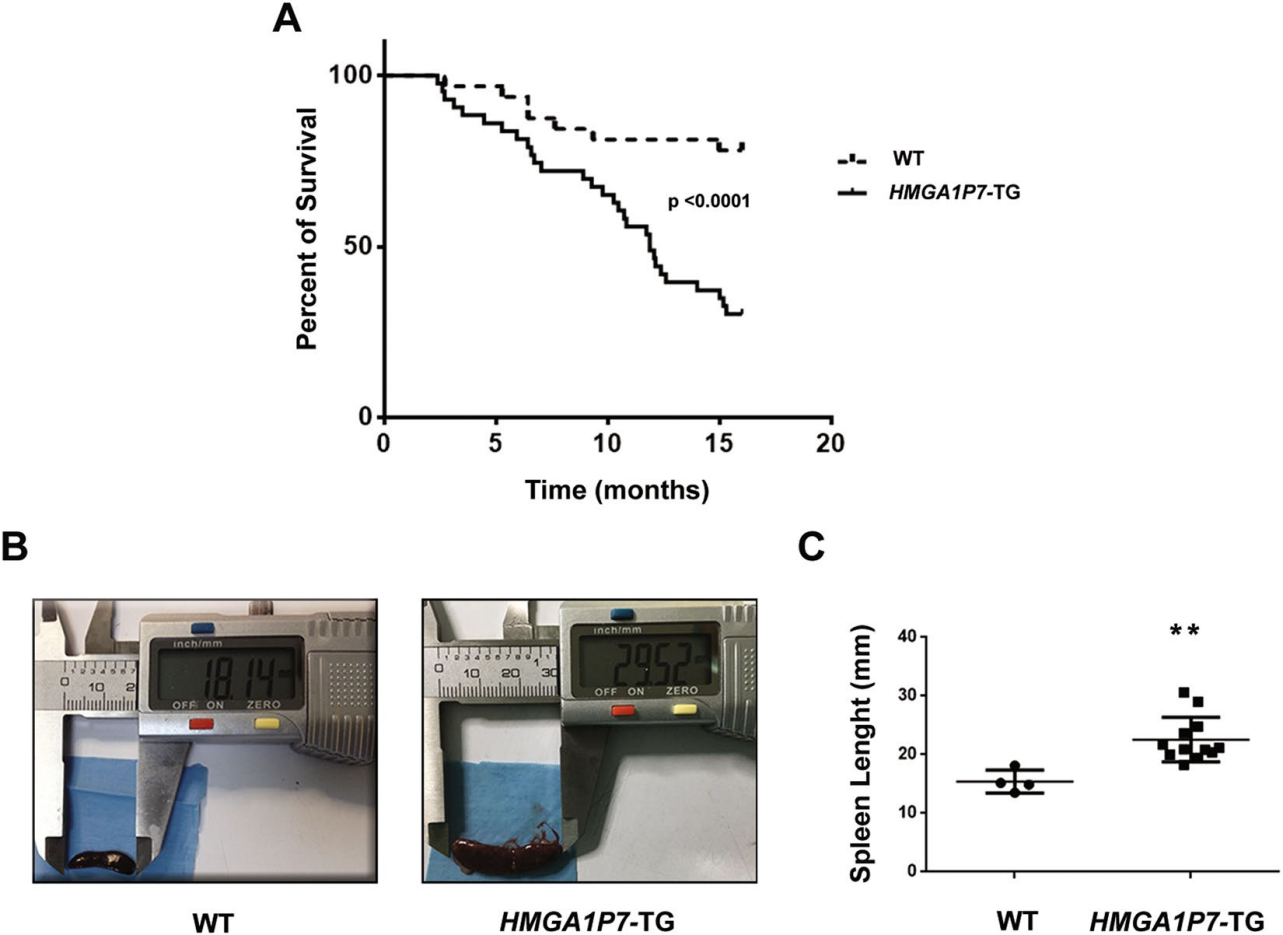
*HMGA1P7* expression *in vivo* induces splenomegaly and premature death (**A**) Survival curve of WT (n = 30) and *HMGA1P7* (n = 40) transgenic mice. The survival rate of WT mice was significantly higher than *HMGA1P7* transgenic ones (Gehan Breslow Wilcoxon test, p < 0.0001). **(B)** Representative images of spleens from WT and *HMGA1P7* transgenic mice. **(C)** Spleens from *HMGA1P7* (n = 12) transgenic mice were larger than spleens from WT (n = 4) (Mann-Whitney Test, **p < 0.0011). The error bars represent mean ± SD.

**Figure 3:**
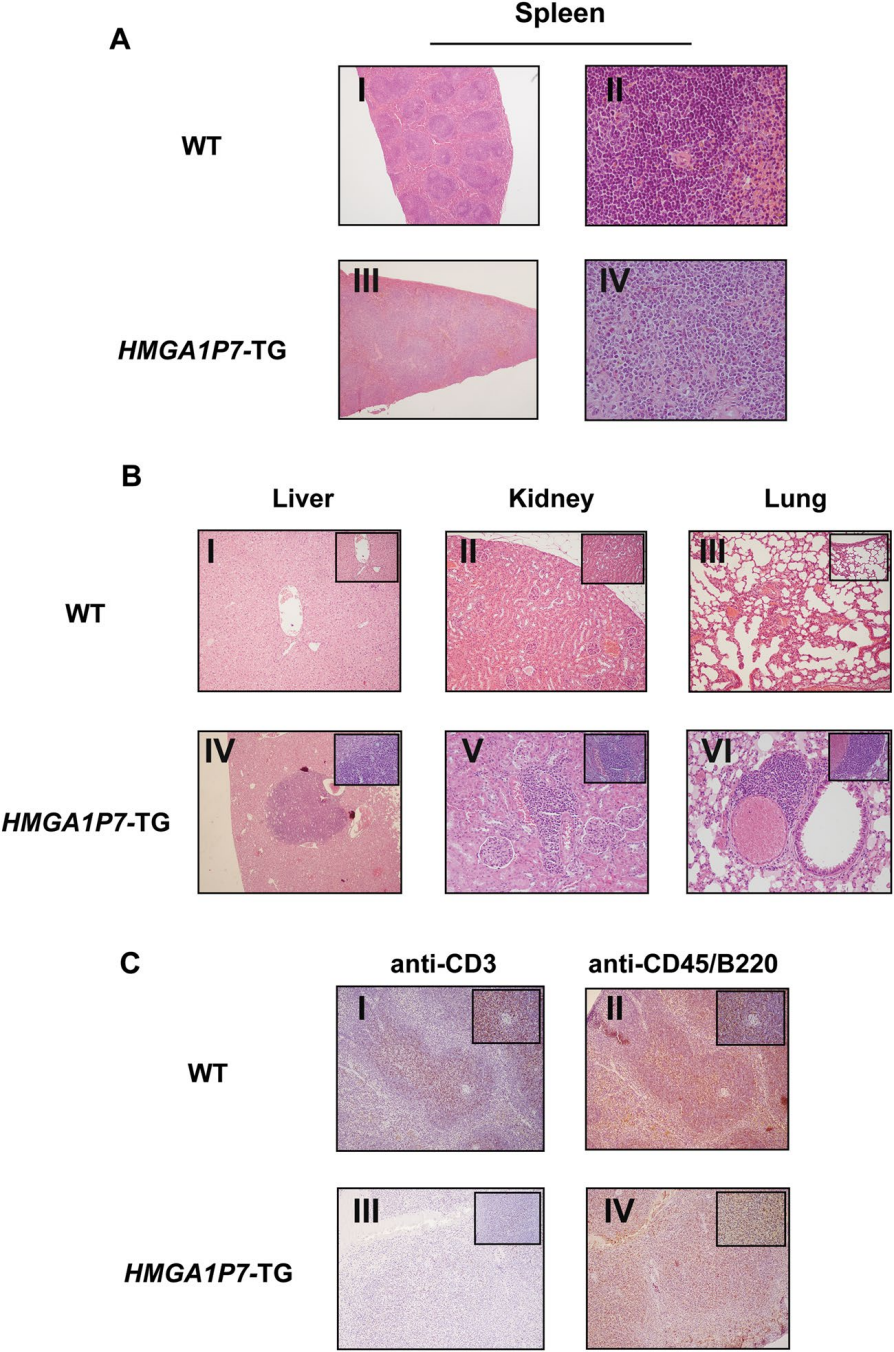
*HMGA1P7* transgenic mice show a lymphoid malignancy (**A**) (I and II) Spleen from WT mouse showing normal morphology. (III) Representative image of immunoblastic lymphoma from a *HMGA1P7*-TG mouse. Expansion and merging of adjacent white pulp areas with loss of normal structures and germinal centers. (IV) A higher magnification shows a monotonous lymphoid population composed of fairly large rounded cells with scant cytoplasm and round to ovalar nuclei with finely dispersed chromatin and inconspicuous nucleoli. Haematoxylin and eosin (Original Magnification 4X for I and III panels, 40X for II and IV panels). **(B)** Representative images of liver (I), kidney (II) and lung (III) from a WT mouse with no pathological alteration. Monotonous lymphoid cells infiltrating the liver (IV), the kidney (V), the lung (VI) of *HMGA1P7*-TG mice. At higher magnification (inset), lymphoid population was composed of fairly large rounded cells with scant cytoplasm and round to ovalar nuclei with finely dispersed chromatin and inconspicuous nucleoli. Haematoxylin and eosin (Original Magnification 10X, inset 40X). (**C**) (I and II) immunohistochemistry of a WT mouse spleen showing normal distribution of CD3 immunolabelled T-cells and CD45/B220 immunolabelled B-cells. (III, IV) Immunohistochemical analysis of *HMGA1P7*-TG mouse spleen revealed a predominant CD45/B220 neoplastic lymphoid population with fewer scattered CD3-immunolabelled cells (Original Magnification 10X, inset 40X).

Interestingly, *HMGA1P7* mice showed significant increased mortality with respect to the WT mice (Gehan Breslow Wilcoxon test, p < 0.0001) with a mean age of death of about 52 weeks (Fig. 2A). About 50% of 12 months-old *HMGA1P7* transgenic mice displayed splenomegaly at necropsy, whereas WT mice showed no relevant alteration in splenic size or weight (Fig. 2B,C). Histological sections of the *HMGA1P7*- TG spleens showed a clear distinction between the red and the white pulp. In the red pulp multiple foci of extramedullary haematopoiesis, as well as hemosiderin-laden macrophages were frequently observed (Fig. 3A,III). White pulp showed a moderate expansion with some confluent areas and partial loss of normal structures and germinal centers. In some mice, higher magnification showed a diffuse, monotonous lymphoid population composed of medium-to-large rounded cells with scant cytoplasm, round to oval nuclei and single or multiple, prominent nucleoli often adherent to the nuclear membrane (Fig. 3A,IV). Mitotic activity was medium to high (<10 × 10 HPF). Intriguingly, histopathological analyses revealed monotonous lymphoid cells infiltrating liver (≈25%), kidneys (≈25%), lung (≈30%), and pancreas (≈20%) (Fig. 3B). Immunohistochemical analysis of lymphoid component displayed a predominant CD45/B220-positive population intermingled with few, scattered CD3-positive cells (Fig. 3C). Based on morphology and immunophenotype, a diagnosis of large B-cell lymphoma with immunoblastic features was made (human counterpart: DLBCL, immunoblastic variant)^25^.

**Figure 4:**
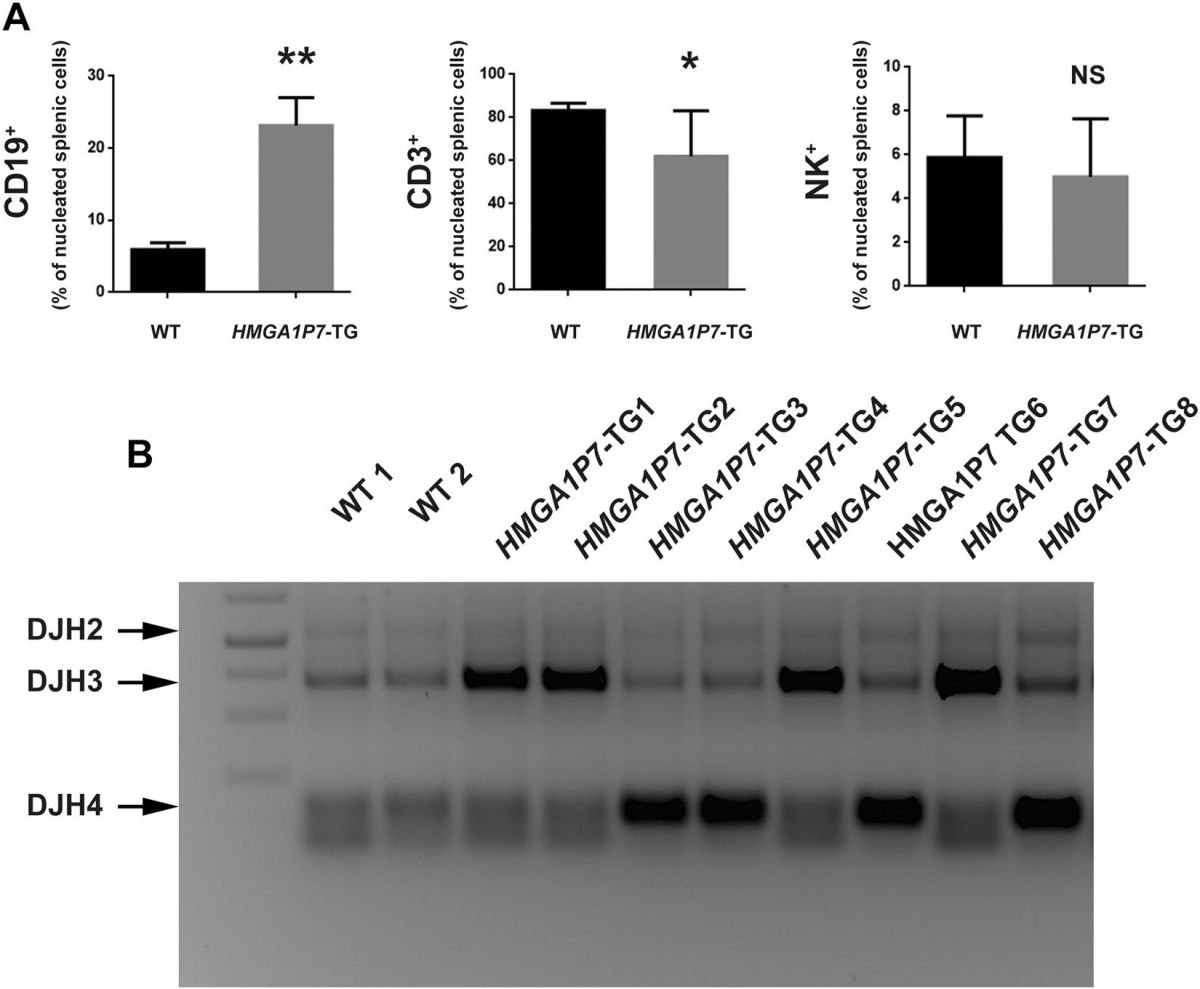
Mice overexpressing *HMGA1P7* develop monoclonal expansion of the CD19 positive population. **(A)** FACScan analysis of splenic cells isolated from WT (n = 8) and *HMGA1P7* (n = 14) transgenic mice using CD19, CD3, and NK1.1 anti-mouse antibodies. The results are reported as the mean of values. The error bars represent mean ± SD; *P < 0.05 **P < 0.01 (t test). **(B)** Genomic DNA isolated from the spleens of two WT mice and eight *HMGA1P7*-TG derived-lymphomas were amplified with DSF and JH4 primers. Three expected DJH bands with the spleen DNA were indicated. Within all eight tumor samples, DJH3 or DJH4 bands were predominantly detected.

Furthermore, FACScan analysis of lymphocytes isolated from WT or pathological spleens using the CD3, CD19 and NK anti-mouse antibodies confirmed the immunohistochemical data. CD19 population resulted almost doubled, while CD3 population was decreased in *HMGA1P7*-TG mouse spleens in comparison with WT animals (Fig. 4A).

To investigate the clonal status of the accumulation of the CD19 positive population in *HMGA1P7*-TG mice, genomic DNAs from TG and WT spleens were analysed. As shown in Fig. 4B, only one dominant PCR product was generated by the amplification of the DNA extracted from the transgenic spleens, whereas DNA derived from a WT spleen yielded three prominent PCR products of 1.0, 0.7 and 0.12 kb, corresponding to DJH2, DJH3 and DJH4 Immunoglobulin (Ig) gene rearrangements, respectively^26^.

Taken together, these results indicate that *HMGA1P7*-TG mice lymphoid expansion was monoclonal, therefore further supporting the diagnosis of B-cell lymphoma.

### Identification of the genes modulated by *HMGA1P7* expression in pathological spleens

**Figure 5:**
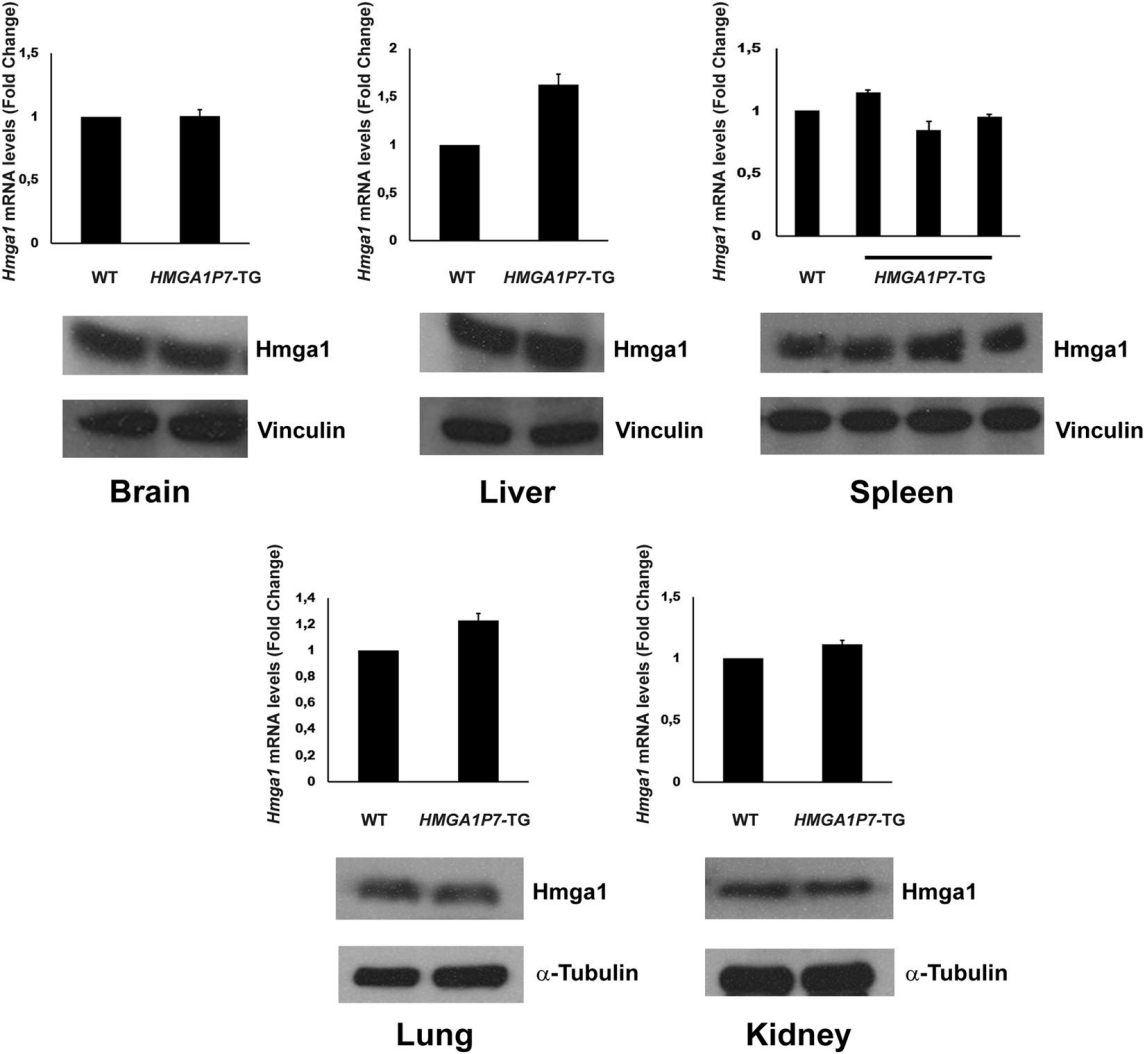
HMGA1 is not upregulated in *HMGA1P7-*TG explanted-organs. qRT-PCR and Western Blot analyses of HMGA1 expression levels in WT (n = 3) and *HMGA1P7* (n = 3) transgenic brain, liver, spleen, lung and kidney organs.

**Figure 6:**
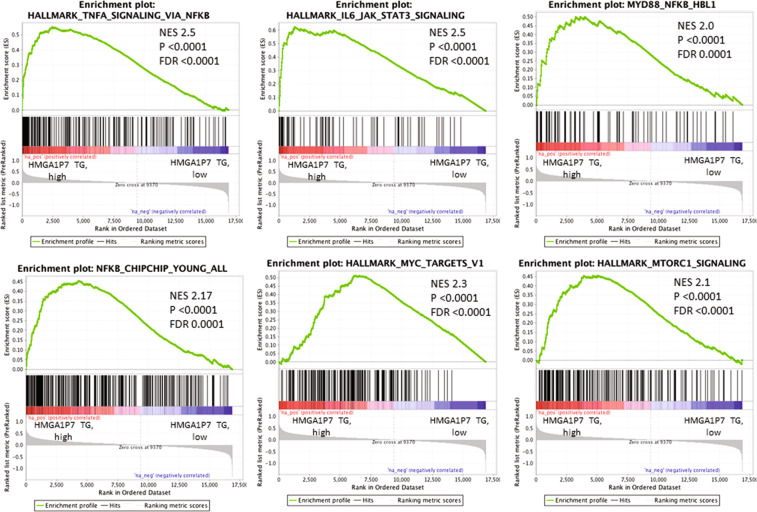
Transcriptome of *HMGA1P7*-TG derived-lymphomas. Representative GSEA plots illustrating the transcriptional expression signature enrichment in genes upregulated in transgenic spleens compared to normal spleens. Green line, enrichment score; bars in the middle portion of the plots show where the members of the gene set appear in the ranked list of genes. Positive or negative ranking metric indicate respectively correlation or inverse correlation with the profile. NES, normalised enrichment score. P, p-value. FDR, false discovery rate.

Since HMGA1 did not result upregulated by *HMGA1P7* overexpression in the analyzed pathological spleens and other mouse tissues (Fig. 5), we compared the transcriptome of spleens derived from *HMGA1P7* transgenic mice (n = 2) *versus* that of WT spleens (n = 2) by RNA-Seq analyses, in order to better understand the mechanisms leading to lymphoid cell proliferation in transgenic mice. The upregulated transcripts included genes involved in inflammation (*Ccl24*, *Il1a*, *Rgs16*, *Ccl5*)^27–30^, in the NFKB pathway and in IL6/JAK/STAT3 and MTOR signalling, in oxidative phosphorylation (*Uqcrc1*, *Ndufa1*, *Cox5a*, *Atp5d*)^31–34^ and targets of MYC, E2F, STAT3, AP1, ATF3. In addition, the spleens of transgenic mice presented a gene expression signature compatible with an induction of senescence (*Il13ra2*, *Il1a*, *Mmp3, Il1b*)^35–38^ and immune escape (*Pvrl2*, *Il10*, *Cd160*, *Ido1*)^39–42^. Enrichment of genes downregulated by B cell receptor (BCR) inhibitors in diffuse large B cell lymphomas (DLBCL) was also unveiled by this analysis. Moreover, among the genes that showed a decreased expression in the transgenic spleens we found (i) transcripts down-regulated in post- germinal center (GC) BCL6 dependent B cell lymphomas when compared to MYC driven pre-GC lymphomas (*Cnot6l, Sh3kbp1)*^43,44^, (ii) genes repressed by BLIMP1 (*Stat6*, *Zfp36l1*)^45,46^; (iii) genes present in the GC B-cell type (GCB) DLBCL signature (*Dtx1,Cux1, Sh3pxd2a, Klhl6*)^47–49^ (Fig. 6).

**Figure 7:**
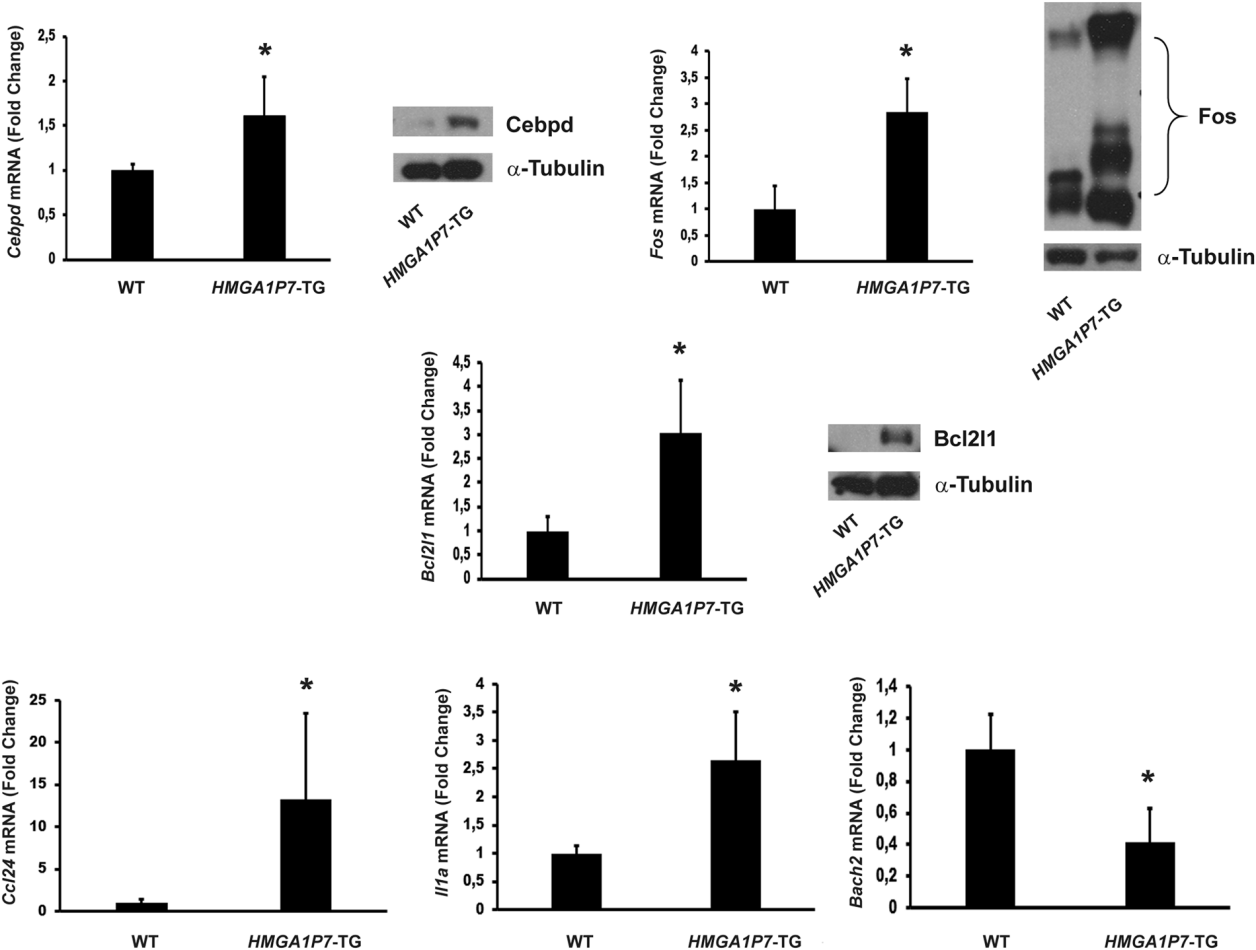
Validation of RNA-Seq analyses on *HMGA1P7* spleens. qRT-PCR and Western Blot analyses of selected deregulated genes performed on WT (n = 4) and *HMGA1P7* (n = 4) transgenic spleens. The results are reported as the mean of values. The error bars represent mean ± SD; *P < 0.05 (Mann-Whitney Test).

**Figure 8:**
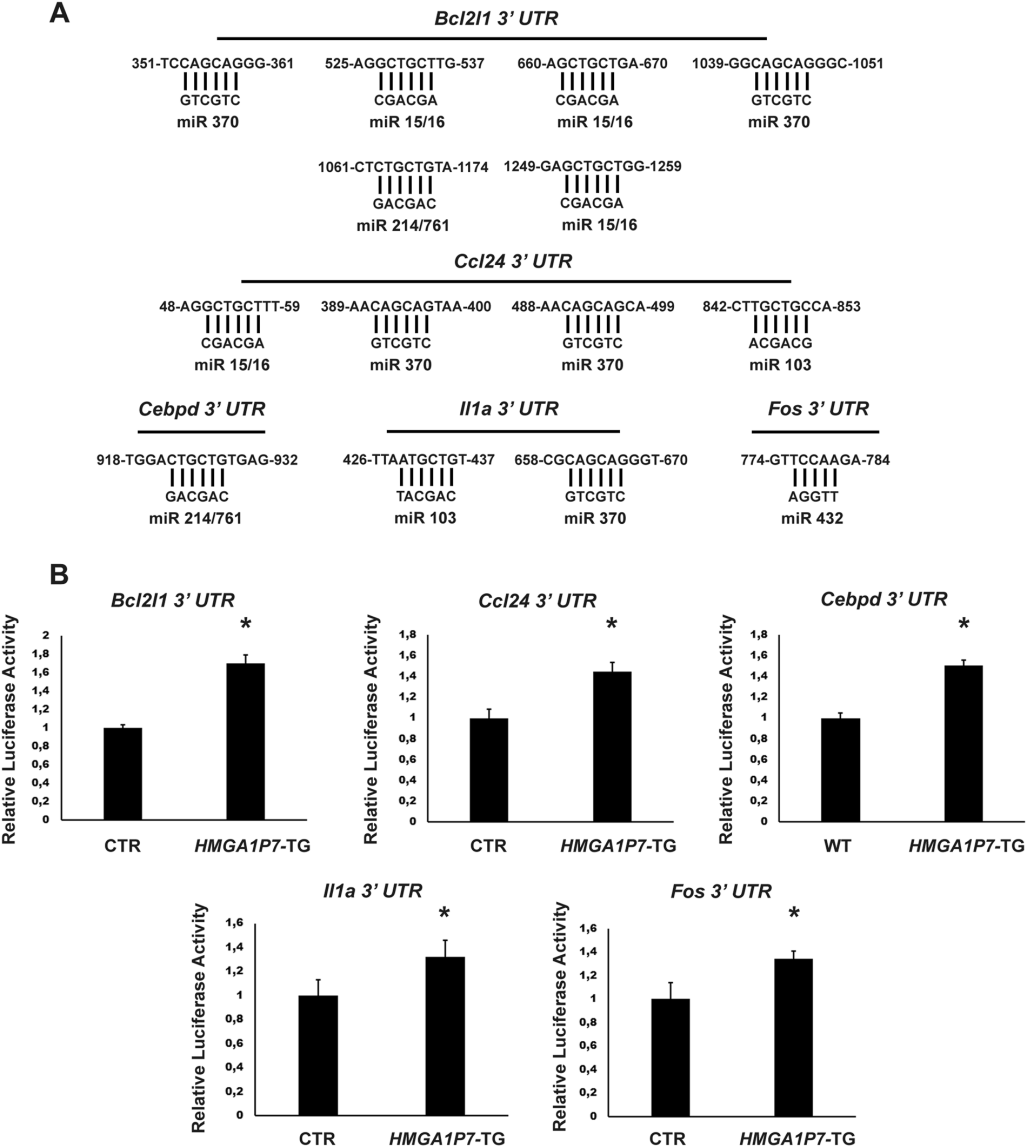
Deregulated genes from RNA-Seq are modulated by *HMGA1P7* through a ceRNA mechanism. (A) 3′UTRs of *Bcl2l1, Ccl24, Cebpd, Il1a* and *Fos* share several microRNA Responsive Elements with *HMGA1P7*. **(B)** The *Bcl2l1, Ccl24, Cebpd, Il1a* and *Fos* 3′UTRs were cloned into the pMIR control vector and then transfected in NIH3T3 cells stably overexpressing the control vector or *HMGA1P7*. The results are reported as the mean of values. Error bars represent mean ± SD; n = 4. *P < 0.05 (Mann-Whitney Test).

Then, we validated the results obtained by RNA-Seq analyses, testing the expression of a panel of deregulated mRNAs in spleens from *HMGA1P7* by qRT-PCR (Fig. 7). Among the upregulated genes we chose CCAAT/enhancer-binding protein delta (*Cebpd*), chemokine (C-C motif) ligand 24 (*Ccl24*), Bcl-2-like 1 (*Bcl2l1*), *Fos*, Interleukin 1 Alpha (*Il1a*), BTB and CNC homolog 2 (*Bach2)*, one of the downregulated genes. Next, the increased expression levels of *Cebpd*, *Bcl2l1* and *Fos* were also confirmed by western blot analyses (Fig. 7). Finally, to demonstrate that *HMGA1P7* acts through a ceRNA mechanism on the genes deregulated in pathological spleens (Fig. 8A), we inserted downstream of the luciferase open reading frame the 3′-UTRs of these genes. These reporter vectors were transfected into NIH3T3 cells overexpressing or not *HMGA1P7*. As expected, the luciferase activity was markedly increased in the cells that overexpressed *HMGA1P7* (Fig. 8B), confirming the ceRNA action induced by *HMGA1P7* on these new targets.

Therefore, on the basis of the FACS and immunohistochemical data combined with the RNA-Seq analyses we can assess that the lymphoproliferation in the *HMGA1P7* transgenic mice shares transcriptome features with DLBCL of the non-GCB type^43–49^.

## Discussion

We have previously reported that the overexpression of *HMGA1Ps* accelerates cell proliferation, by enhancing the G1-S transition, increases cell migration ability, likely raising the levels of HMGA1 and other oncogenic proteins such as HMGA2 and EZH2^18^. Moreover, the MEFs obtained from *HMGA1Ps* transgenic mice showed a reduced proliferation time and senescence in comparison with the WT MEFs^18^.

Therefore, the aim of this study was to better characterize the transgenic mice overexpressing the *HMGA1P7* to possibly validate its oncogenic activity *in vivo*. The analysis of *HMGA1P7* transgenic mice at 12 months of age, shows that about 50% of these mice developed a pathology characterized by splenomegaly and invasion of lymphoid cells in different anatomical districts. The pathological spleens showed a diffuse and monotonous lymphoid population effacing the splenic parenchyma with the loss of typical structures and germinal centres. Neoplastic lymphoid cells were medium to large, rounded, with scant cytoplasm and round to ovular nuclei with single or multiple prominent nucleoli.

By immunohistochemistry and FACS analyses, we found that the neoplastic cells were respectively positive for CD45/B220 and CD19 proposing a B cell phenotype of the lymphoid cells. Clonality assay on pathological spleens evidenced the clonal expansion of CD19-positive lymphoid population supporting a diagnosis of B cell lymphomas for these lesions. Interestingly, RNA-Seq analyses performed on spleens derived from WT and *HMGA1P7* mice revealed a deregulation of several genes, likely due to *HMGA1P7-*ceRNA activity. The deregulated genes were involved in inflammation pathways such as NFKB pathway, the IL6/JAK/STAT3 and MTOR signalling, the oxidative phosphorylation, and targets of MYC, E2F, STAT3, AP1, ATF3. Moreover, the spleens from *HMGA1P7* mice had a gene expression signature compatible with an induction of senescence and immune escape (*Il13ra2*, *Il1a*, *Mmp3, Il1b, Pvrl2*, *Il10*, *Cd160*, *Ido1*)^35–42^.

Noteworthily, the genes suppressed by BCR inhibitors in DLBCL were found significantly enriched in the pathological tissues of *HMGA1P7* mice. In particular, the downregulated genes were enriched of transcripts decreased in post-GC BCL6 dependent B cell lymphomas and present in the GCB DLBCL signature. Therefore, the transcriptome study of the lymphoproliferative lesions in the *HMGA1P7* transgenic mice unveils a pathology compatible with DLBCL of the non-GCB type.

Consistently with the ability of the *HMGA1Ps* to regulate gene transcription by a ceRNA mechanism, bioinformatic analyses demonstrate that several upregulated genes emerged from RNA-Seq data shared the same microRNA Responsive Elements with *HMGA1P7* (*i.e. Cebpd*, *Ccl24*, *Bcl2l1*, *Fos*, *Il1a*).

Surprisingly, HMGA1 did not result upregulated by *HMGA1P7* overexpression in the analysed pathological spleens, suggesting that pseudogene-induced lymphomas were based on other molecular targets already described^27–49^. Moreover, we did not find any change in HMGA1 expression levels during spleen development of *HMGA1P7*-transgenic mice (data not shown). However, we cannot exclude the possibility of increased HMGA1 protein levels in a limited cell compartment in the initial steps of lymphomagenesis.

Altogether the data presented here show that deregulated expression of *HMGA1P7* pseudogene has oncogenic role also *in vivo*, thus representing a new class of genes involved in cancer pathology as their upregulation occurs frequently in multiple human cancers^50^. An oncogenic role for pseudogenes has been already reported. Indeed, mice engineered to overexpress the full-length murine *B-Raf* pseudogene *Braf-rs1* develop an aggressive malignancy resembling human diffuse large B cell lymphoma by ceRNA mechanism that elevates *BRAF* expression^50^.

Noteworthy, preliminary studies on a mouse strain overexpressing *HMGA1P6* pseudogene show that several mice develop a lymphoid pathology characterized by splenomegaly that resembles that found in *HMGA1P7*-TG mice.

Therefore, our mouse model confirms the oncogenic potential of pseudogenes and provides compelling support for a causal link between altered pseudogene expression and cancer, mediated by ceRNA mechanism. Studies are in progress to evaluate the expression levels of *HMGA1* pseudogenes in human lymphomas. Preliminary results indicate *HMGA1P1* overexpression that could contribute to lymphomagenesis by a similar ceRNA mechanism.

## Materials and Methods

### Transgenic mice

*HMGA1P7* overexpressing mice have been previously generated and described^18^. Animals were housed in IRCCS “G. Pascale” animal facility as previously reported^51^ (project “Ruolo degli pseudogeni di HMGA1 nel cancro” Cod. 893/2013 approved by Italian Health Ministry on 13/05/2013). The experimental protocols were in complaints with the European Communities Council Directive (63/2010/EEC).

### Cell culture and transfections

NIH3T3 cells were maintained in DMEM supplemented with 10% calf serum (Thermofisher, Waltham, MA, USA), glutamine and antibiotics. MycoAlert (Basel, Switzerland) was regularly used to test that cells were not infected with mycoplasma. Cell transfection protocol was reported elsewhere^19^.

## Histology and immunohistochemistry

Light microscopy was performed as previously described^52^. Definition and classification of lymphoid disease were based on criteria reported elsewhere^25^.

For immunohistochemistry (IHC), 4-μm-thick sections were processed as previously described^24,53^. Primary antibodies included rabbit monoclonal to CD3 (ab16669, Abcam, Cambridge, UK) diluted 1: 200 and rabbit polyclonal to CD45/B220 as a marker for B cells^54^ (ab10558 Abcam, Cambridge, UK) diluted 1:1000.

## RNA extraction and qRT-PCR

RNA extraction, RNA reverse transcription and Real-time PCR was performed as described elsewhere^55^. The following primers were used:

HMGA1P7-Fw 5′-gctccttctcggctcctc-3′HMGA1P7-Rev 5′-gcttgggcctcttttatgg-3′

Hmga1-Fw 5′-ggcagacccaagaaactgg-3′Hmga1-Rev 5′-ggcactgcgagtggtgat-3′

Cebpd-Fw λ5′-cttttaggtggttgccgaag-3′Cebpd-Rev 5′-ggcaacgaggaatcaagttt-3′

Ccl24-Fw 5′-gcagcatctgtcccaagg-3′Ccl24-Rev 5′-gcagcttggggtcagtaca-3′

G6pd-Fw 5′-cagcggcaactaaactcaga-3′G6pd-Rev 5′-ttccctcaggatcccacac-3′

Bcl2l1-Fw 5′-tgaccacctagagccttgga-3′Bcl2l1-Rev 5′-gctgcattgttcccgtaga-3′

Fos-Fw 5′-gggacagcctttcctactacc-3′Fos-Rev 5′-agatctgcgcaaaagtccg-3′

Il1a-Fw 5′-ttggttaaatgacctgcaaca-3′Il1a-Rev 5′-gagcgctcacgaacagttg-3′

Bach2-Fw 5′-gcagacagtgagtcgtgtcc-3′Bach2-Rev 5′-gttcctgggaaggtctgtga-3′

## Flow cytometric analysis (FACS)

For FACS analyses, spleens were collected from WT and transgenic mice, hard-pressed through a stainless-steel mesh, resuspended in PBS and then in Red Blood Lysing Buffer (Sigma-Aldrich, Saint Louis, MI, US) for 3 min. After two washes in PBS, lymphocytes (5 × 10^5^) were set in 96-well round-bottom dishes.

Monoclonal antibodies utilized: NK-FITC (clone # PK146), CD3 APC-H7 (clone # 560176), CD19 PE/cy7 (clone # HIB19). All antibodies were from BioLegend (San Diego, CA, US).

## Analysis of the clonality of lymphomas

Genomic DNA was extracted from fresh spleens through Phenol/Chloroform/Isoamyl Alcohol Extraction (Thermofisher, Waltham, MA, USA). The obtained DNAs were utilized as PCR templates with DSF and JH4 primers that recognize mouse DNA DJ rearrangement^26^.

DSF primer: 5′-AGGGATCCTTGTGAAGGGATCTACTACTGTG-3′;

JH4 primer: 5′-AAAGACCTCCAGAGGCCATTCTTACC-3′.

## RNA-Seq analyses

Genomix4life S.R.L. (Baronissi, Salerno, Italy) performed the next generation sequencing analysis, including samples quality control and Bioinformatics analysis. Following the producer’s guidelines, indexed libraries were obtained from 500 ng/ea RNA through TruSeq Stranded total RNA Sample Prep Kit (Illumina, San Diego, CA, USA). The libraries quantification was performed by the Agilent 2100 Bioanalyzer (Agilent Technologies, Santa Clara, CA, USA) and Qubit fluorometer (Thermofisher, Waltham, MA, USA), then combined in order that every index-tagged sample was in equimolar amounts, with 2 nM pooled samples final concentration. Illumina HiSeq. 2500 System (Illumina, San Diego, CA, USA) sequenced the pooled samples with a format of 2 × 100 paired-end at 8 pmol final concentration.

FastQC tool^56^ was utilized for the quality control analysis of the generated raw sequence files (.fastq files). Cutadapt was used in order to eliminate the adapter sequences. Paired-end reads were mapped using STAR (version 2.5.2b)^57^ on reference genome assembly mm10 acquired from Ensembl^58,59^. The quantification of transcripts expressed for each replicate of the sequenced samples was performed using HTSeq-Count algorithm^60^. The differential expression analysis was performed through DESeq. 2^61^.

Gene Set Enrichment Analysis (GSEA) was used for functional annotation on pre-ranked lists using the MSigDB 5.2^62^, the SignatureDB collection^63^ and genesets obtained from different publications^64,65^, applying false discovery rate (FDR) values <0.05 as threshold.

## Luciferase assay

Dual-luciferase reporter assays were performed as previously described^18^.

## Plasmids

For the mCebpd (NM_007679), mCcl24 (NM_019577), mBcl2l1 (NM_009743), mFos (NM_010234) and mIl1a (NM_010554) 3′UTR luciferase reporter constructs, the 3′UTR sequences were amplified by using the following primers:

mCebpd 3′utr-Fw 5′-gcagagctcagaattctgcctttctactaagatactggttg-3′

mCebpd 3′utr-Rv 5′-gcgatcgcttgaattcttagtgttctgggagctgcc-3′

mCcl24 3′utr-Fw 5′-gcagagctcagaattcccgcctctcctctgtccc-3′

mCcl24 3′utr-Rv 5′-gcgatcgcttgaattcacatcctggcagcaagagg-3′

mBcl2l1 3′utr-Fw 5′-gcagagctcagaattcgagcctctcgggaatgcttttc-3′

mBcl2l1 3′utr-Rv 5′-gcgatcgcttgaattccgcacagcaagccagcag-3′

mFos 3′utr-Fw 5′-gcagagctcagaattcgaatgttctgacattaacagttttc-3′

mFos 3′utr-Rv 5′-gcgatcgcttgaattcttcaacttaaatgcttttattgac-3′

mIl1a 3′utr-Fw 5′-gcagagctcagaattccaaaatgccagttgagtagga-3′

mIl1a 3′utr-Rv 5′-gcgatcgcttgaattcaggagactacatctaactgaccac-3′

The amplified fragments were cloned into pMirTarget vector (OriGene, Rockville, MD, USA) using In-Fusion HD Cloning kit (Takara Bio, Mountain View, CA, USA). *HMGA1P7* overexpressing vector was previously described^18^.

## Western blot

Western blot analyses were performed as previously described^66^. The primary antibodies used were: anti-Cebpd #7077 (ProSci, Poway, CA, USA); anti-Fos sc-166940 (Santa Cruz Biotechnology, Dallas, TX, USA); anti-Bcl2l1 #2762 (Cell Signaling, Danvers, MA, USA). Antibody against HMGA1 protein was described elsewhere^67^.

## Statistical analysis

Two-sided unpaired Student’s t tests and Mann-Whitney tests were utilized to analyse data (GraphPad Prism, GraphPad Software, Inc.). P < 0.05 values were taking into account as statistically significant. The mean values +/− s.d were obtained from three or more separate experiments. GraphPad Prism, GraphPad Software, Inc. was used to obtain regression analyses and correlation coefficients.
