# Publisher Correction: *HMGA1-pseudogene7* transgenic mice develop B cell lymphomas

**DOI:** 10.1038/s41598-021-92173-4

**Published:** 2021-06-11

**Authors:** Marco De Martino, Davide De Biase, Floriana Forzati, Sara Carmela Credendino, Giuseppe Palma, Antonio Barbieri, Claudio Arra, Orlando Paciello, Eugenio Gaudio, Maurilio Ponzoni, Gabriella De Vita, Paolo Chieffi, Francesco Bertoni, Alfredo Fusco, Francesco Esposito

**Affiliations:** 1 Istituto di Endocrinologia ed Oncologia Sperimentale - CNR c/o Dipartimento di Medicina Molecolare e Biotecnologie Mediche, Università degli Studi di Napoli “Federico II”, Naples, Italy; 2 Department of Veterinary Medicine and Animal Production, University of Naples Federico II, Naples, Italy; 3 S.S.D. Sperimentazione Animale, Istituto Nazionale Tumori, IRCCS, Fondazione Pascale, Naples, Italy; 4 Institute of Oncology Research, Faculty of Biomedical Sciences, USI, Bellinzona, Switzerland; 5 Vita-Salute San Raffaele University & Pathology Unit, IRCCS San Raffaele Scientific Institute, Milan, Italy; 6 Department of Psychology, University of Campania “L. Vanvitelli”, Caserta, Italy

Correction to: *Scientific Reports* https://doi.org/10.1038/s41598-020-62974-0, published online 27 April 2020

In the original version of this Article, the author Gabriella De Vita was incorrectly indexed. This error has now been corrected.

